# Protocol paper: kainic acid excitotoxicity-induced spinal cord injury paraplegia in Sprague–Dawley rats

**DOI:** 10.1186/s40659-022-00407-0

**Published:** 2022-12-09

**Authors:** Anam Anjum, Yt Jun Cheah, Muhammad Da’in Yazid, Muhammad Fauzi Daud, Jalilah Idris, Min Hwei Ng, Amaramalar Selvi Naicker, Ohnmar Htwe Ismail, Ramesh Kumar Athi Kumar, Geok Chin Tan, Yin Ping Wong, Mohd Kaisan Mahadi, Yogeswaran Lokanathan

**Affiliations:** 1 Centre for Tissue Engineering and Regenerative Medicine, Faculty of Medicine, Universiti Kebangsaan Malaysia, Jalan Yaacob Latif, Cheras, 56000 Kuala Lumpur, Malaysia; 2 Department of Physiology, Faculty of Medicine, Universiti Kebangsaan Malaysia, Jalan Yaacob Latif, Cheras, 56000 Kuala Lumpur, Malaysia; 3 Institute of Medical Science Technology, Universiti Kuala Lumpur Malaysia, 43000 Kajang, Selangor Malaysia; 4 Department of Orthopaedics & Traumatology, Faculty of Medicine, Universiti Kebangsaan Malaysia, Jalan Yaacob Latif, Cheras, 56000 Kuala Lumpur, Malaysia; 5 Department of Surgery, Faculty of Medicine, Universiti Kebangsaan Malaysia, Jalan Yaacob Latif, Cheras, 56000 Kuala Lumpur, Malaysia; 6 Department of Pathology, Faculty of Medicine, Universiti Kebangsaan Malaysia, Jalan Yaacob Latif, Cheras, 56000 Kuala Lumpur, Malaysia; 7 Drug and Herbal Research Centre, Faculty of Pharmacy, Universiti Kebangsaan Malaysia, Jalan Raja Muda Abdul Aziz, 50300 Kuala Lumpur, Malaysia

**Keywords:** Spinal cord injury (SCI), Kainic acid, Locomotion, Motor neuron, Intra-spinal injection

## Abstract

**Background:**

Excitotoxicity-induced in vivo injury models are vital to reflect the pathophysiological features of acute spinal cord injury (SCI) in humans. The duration and concentration of chemical treatment controls the extent of neuronal cell damage. The extent of injury is explained in relation to locomotor and behavioural activity. Several SCI in vivo methods have been reported and studied extensively, particularly contusion, compression, and transection models. These models depict similar pathophysiology to that in humans but are extremely expensive (contusion) and require expertise (compression). Chemical excitotoxicity-induced SCI models are simple and easy while producing similar clinical manifestations. The kainic acid (KA) excitotoxicity model is a convenient, low-cost, and highly reproducible animal model of SCI in the laboratory. The basic impactor approximately cost between 10,000 and 20,000 USD, while the kainic acid only cost between 300 and 500 USD, which is quite cheap as compared to traditional SCI method.

**Methods:**

In this study, 0.05 mM KA was administered at dose of 10 µL/100 g body weight, at a rate of 10 µL/min, to induce spinal injury by intra-spinal injection between the T12 and T13 thoracic vertebrae. In this protocol, detailed description of a dorsal laminectomy was explained to expose the spinal cord, following intra-spinal kainic acid administration at desired location. The dose, rate and technique to administer kainic acid were explained extensively to reflect a successful paraplegia and spinal cord injury in rats. The postoperative care and complication post injury of paraplegic laboratory animals were also explained, and necessary requirements to overcome these complications were also described to help researcher.

**Results:**

This injury model produced impaired hind limb locomotor function with mild seizure. Hence this protocol will help researchers to induce spinal cord injury in laboratories at extremely low cost and also will help to determine the necessary supplies, methods for producing SCI in rats and treatments designed to mitigate post-injury impairment.

**Conclusions:**

Kainic acid intra-spinal injection at the concentration of 0.05 mM, and rate 10 µL/min, is an effective method create spinal injury in rats, however more potent concentrations of kainic acid need to be studied in order to create severe spinal injuries.

**Supplementary Information:**

The online version contains supplementary material available at 10.1186/s40659-022-00407-0.

## Introduction

**Fig. 1:**
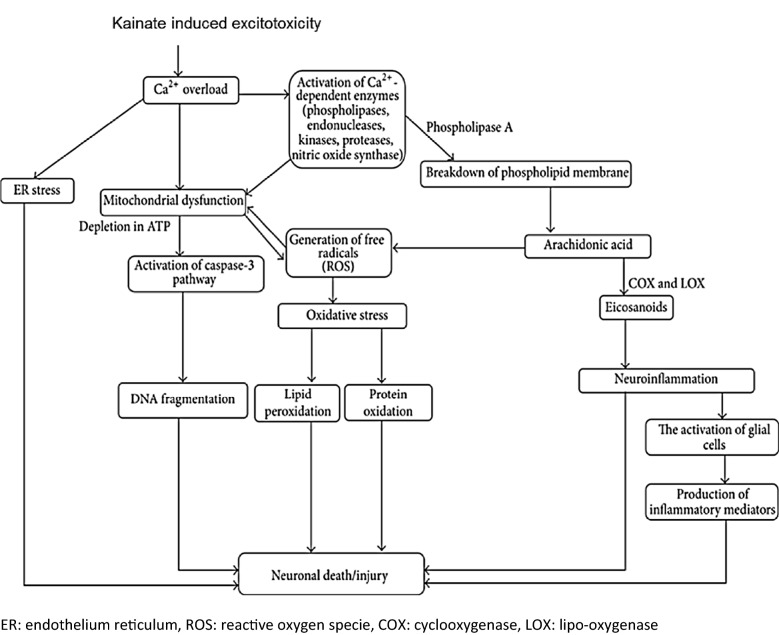
Proposed mechanism of action in KA-induced excitotoxicity

In vivo spinal cord injury (SCI) models are considered non-replaceable as they can relate to similar pathophysiological conditions in humans. SCI models aid understanding of the injury mechanisms and benefit analysis of advanced therapeutic interventions [1]. Due to their close analogy in the functional, morphological, and electrophysiological consequences of SCI in humans, the most commonly utilised animals for studying various neuronal pathological conditions are rodents, including rats and mice [2]. SCI is classified either as complete or incomplete injury. A complete SCI describes the complete loss of sensation and muscle function at and below the injury site. An incomplete SCI refers to partial function loss below the injury level [3]. The level of injury is another crucial aspect: SCI in the cervical and upper thoracic region can cause inconsistent breathing patterns and lead to death. Injury in the lower thoracic or lumbar region is preferable as an SCI model as it only produces paraplegia without altering respiratory and cardiac functions [3]. SCI models are categorised based on the mechanism of injury: mechanical or chemical. Mechanical injury is caused by mechanical means such as impactors, forceps, clips, balloons, or scissors [4] while chemical injury is caused by injecting chemicals such as glutamate, aspartate, *N*-methyl-d-aspartate (NMDA), superoxide, hydroxyl radical and peroxynitrate, heavy metals, ethidium bromide, or kainate [5]. There are various mechanical injury models for producing complete and incomplete SCI, such as contusion, compression, distraction, dislocation, or transection [1]. The mechanical injury model is advantageous for assessing axonal regeneration and subsequent functional recovery [5]. The chemical injury model is useful for investigating axonal and neuronal degeneration, molecular mechanisms, and the effect of various therapies on specific pathways [5]. The excitotoxic chemical injury model is gaining popularity [6] as it is useful for studying secondary injury mechanism events such as neuronal and axonal degeneration caused by glutamate excitotoxicity [7]. Neurodegeneration is described as the progressive loss of structure and function of neurons, axons, and nerve cells [8]. Chronic neurodegenerative diseases, such as Parkinson disease (PD), Huntington disease [1], Alzheimer disease (AD) [9], temporal lobe epilepsy, and amyotrophic lateral sclerosis [10] occur because of chemical excitotoxicity [6]. 6-Hydroxydopamine (6-OHDA) causes neurotoxicity that produces PD [10] and G93A mutation causes the hydroxyl radical production in transgenic ALS rats, which further implicates oxidative damage causing ALS pathogenesis [11]. The exposure of the brain and spinal cord to other chemicals, such as heavy metals (aluminium) cause cognitive impairment and generally damage the nervous system [9], while scopolamine causes dementia [9], colchicine induces AD symptoms [9], and kainic acid (KA) produces temporal lope epilepsy via intrahippocampal or intra-amygdaloid administration [11] or SCI via intra-spinal administration [12].

l-Glutamate is an excitatory transmitter involved in learning, cognition, memory, endocrine functions, and neuron activity. There are two types of glutamate receptors: Ionotropic and metabotropic. Ionotropic receptors are involved in excitotoxicity through AMPA (α-amino-3-hydroxy-5-methyl-4-isoxazolepropionic acid), kainate, and NMDA receptor activation [3]. Excessive glutamate is highly toxic to neurons and is known as glutamate excitotoxicity [6]. Glutamate excitotoxicity neuronal injury models are simple and reliable. Glutamate excitotoxicity is triggered primarily due to excessive Ca^2+^ influx, endoplasmic reticulum (ER) membrane disintegration and ER stress, reactive oxygen species [13] production, mitochondrial dysfunction, neuronal cells apoptosis [3]. A potent agonist of the AMPA/kainate class of glutamate receptors, KA is a non-degradable analogue of glutamate and causes 30 times more potent neurotoxicity than glutamate [11]. In Sprague–Dawley (SD) rats, KA led to pathological conditions such as seizures, brain nerve cell degeneration, and neurodegenerative pathway activation (Fig. 1). KA causes microglial cell, astrocyte, and neuron degeneration. The proposed mechanism of action of KA-induced excitotoxicity is depicted in Fig. 1.

In this article, we detail the procedure for inducing KA excitotoxicity in SD rats by direct injection of KA to the spinal cord to generate a SCI. We also describe the unique concerns regarding the postoperative care of laboratory animals with SCI to improve their quality of life and survival and reduce mortality.

## Materials and equipment

### Surgical instruments

Animal shaver, muscle marker, blades, forceps (blunt-end), forceps (middle-pointed), retractors, scalpel holder, scalpel (size 11), iris scissors (straight), sutures (2/0), needle holder, surgical gauze sponge, syringe (1 mL), needle (26 G), wipes, pillow (for head-up position), surgical paper, autoclave paper bags.

### Equipment

RodVent small-animal ventilator, Opure 10 (10 L portable oxygen cylinder), reptile heating pad (35–40 °C), blanket (to cover the animal), heating water bath, surgical lamp, autoclave, clear Acrylic box (5 mm thickness, 610 mm width and 410 mm height), metal running wheel for pets (32 cm), and stainless steel grid (40 × 60 cm).

### Chemicals

Isopropyl alcohol (70%), ketamine (90 mL), xylazine (10 mL), povidone (10% iodine), antibiotic injection (Baytril (100 mg/mL), tramadol injection (50 mg/mL), Ringer lactate solution, KA (0.1 mM), 0.9% normal saline for injection, wound clear solution, eye ointment and pentobarbital (200 mg/mL).

## Methods

### Animal housing

Keeping the animal’s distress to a minimum level will lead to optimal performance throughout an experimental study and result in both less morbidity and mortality. The animals were kept for 7 days prior to injury to recover from their trip and to acclimate to new housing conditions. Autoclaved drinking water was also provided. The animals were housed individually in single clean cage under bio-bubble air control system and their body weights were measured every day. The animals were allowed ad libitum eating and drinking for 7 days before surgery and then the food and water intake was optimized after SCI. Beside this the animals were undergo a physical examination during these 7 days. Each animal was assessed on these six categories (body weight, physical appearance, behaviour/activity, clinical signs, lesions/autonomy, and bladder functions).

### Surgical preparations

**Fig. 2:**
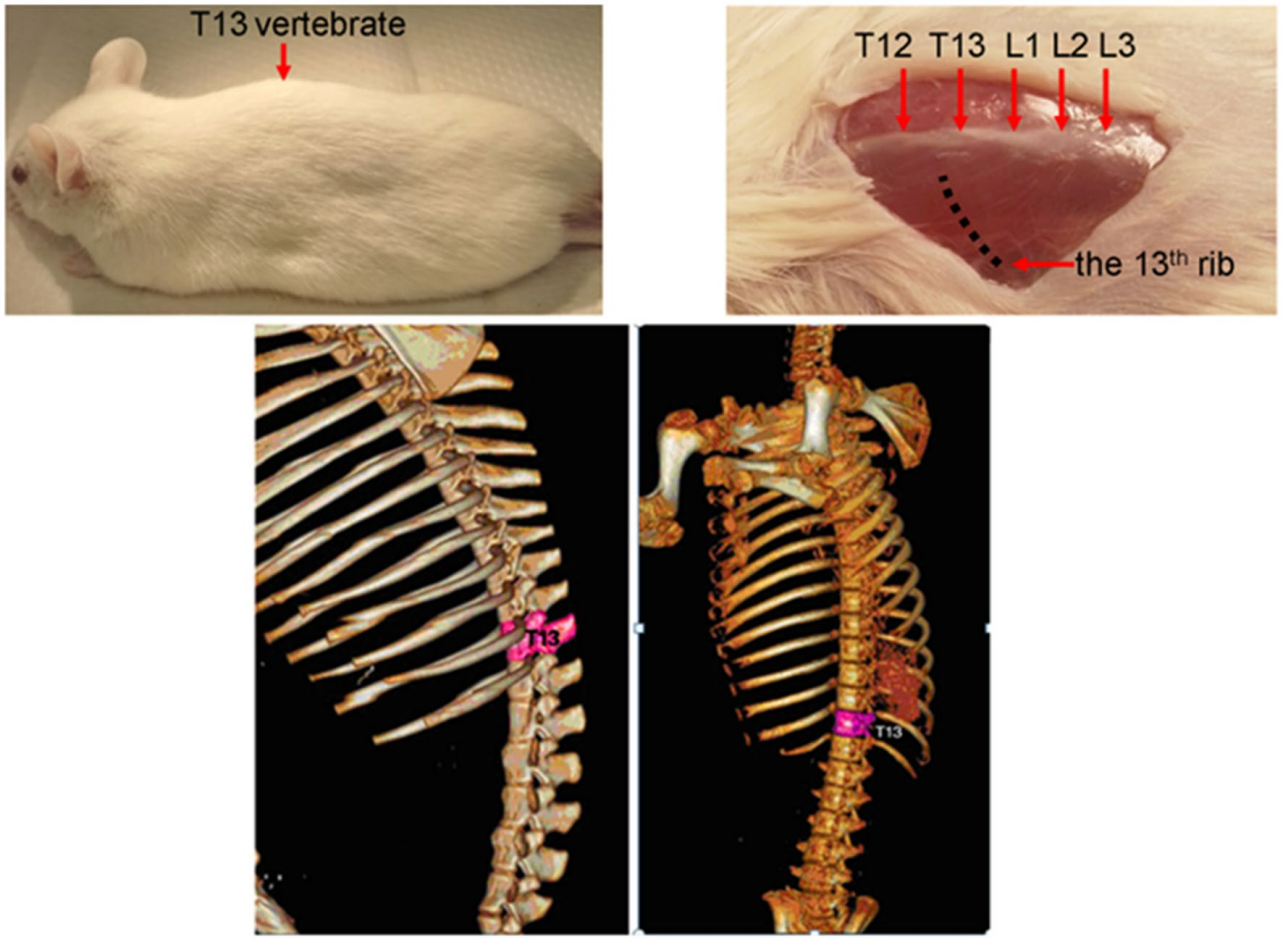
Anatomical location of the T13 thoracic spinal cord segment in a top-down view of the back of the SD rat. The skin was opened to expose the vertebral column at T12–L3. T13 is adjacent to the 13th rib

The surgical tools and surgical paper were autoclaved prior to surgery. Three sets of surgical tools were prepared as more than one surgery per day was planned. The tools were sterilised between surgeries. All necessary surgical tools and chemicals [forceps, iris scissors, retractors, scalpels, scissors, sutures, sterile saline, surgical sponges, povidone solution, eye ointment, and ketamine-xylazine (KTX) cocktail (9:1)] were placed close to the operation table. The operation table, RodVent ventilator, surgical lamp, and heating pad were wiped with 70% isopropyl alcohol before each surgery. A disposable surgical gown, two pairs of surgical gloves, and surgical cap were worn to avoid contamination and to ensure sterility throughout the surgeries. Each rat was carefully weighed prior to surgery; the RodVent setting was adjusted by adding the animal’s weight and then the portable oxygen cylinder was attached to provide enough oxygen during surgery. The temperature of the heating pad and water bath were adjusted to 37 °C, and then KTX solution, normal saline, KA, and Ringer lactate solution were incubated therein to maintain the temperature of the solutions at 37 °C. The adult (n = 10, 300–400 g or 8 week old) specific-pathogen-free (SPF) male Sprawled Dawley rats were obtained from Animal experimental unit (AEU), faculty of medicine Universiti of Malaya. The rats were anesthetised by administering KTX solution via intramuscular injection at 0.1 mL/100 g animal body weight. Once the animal was anesthetised, ointment was applied to the eyes to prevent dehydration, and the animal was placed on the heating pad. The dorsal surface was shaved and the vertebrae were marked from the T11 to T13 positions approximately 1 cm around the intended incision location. The T13 vertebra was determined by palpating the 13th rib externally, then the vertebra was visualised with forceps (the forceps were hooked gently underneath the rib to determine to which vertebra it was attached, i.e. the T13 vertebra will move in response to the rib movement. Then, we counted upward to find the T11 vertebra (Fig. 2). The incision site was disinfected three times with 70% isopropyl alcohol, then with iodine solution.

### Dorsal laminectomy

All the experiments were conducted after getting approval from University Kebangsaan Malaysia, Animal Ethical Committee (UKMAEC) with approval TEC/FP/2021/YOGESWARAN/27-MAY/1176-JUNE-2021-JUNE-2023. Before making the incision, we ensured that the animal was properly anesthetised by checking for reflexes using the toe or tail pinch method. Then, an incision was made along the dorsal spine, and the animal’s reflexes were checked again. A line was drawn along the T11–T13 vertebrae and the skin was cut through approximately 1.5 cm. A skin retractor was inserted to hold the skin and the tissue was cleared on either side of the spinal cord to locate the T13 vertebra. With proper lighting, the space between the T12 and T13 vertebrae was determined (Fig. 4). The needle was slowly inserted between the vertebrae, and reflexes in the lower limb and tail were checked for proper positioning. A pilot study was done comprising of 2 animals in each group i.e., healthy animal, injured animal and sham group (same procedure without KA as a control).

### Administration of KA

The rat was placed in a prone position by placing the pillow under the abdomen to avoid perfusion of the drug towards the brain. The perfusion of KA to the brain tissue will rapidly induce seizure; the reason epileptic seizures follow SCI remains unclear (supplementory video 2). The brain and spinal cord share a common origin, which is supported by the co-expression of specific neurotransmitters in both locations. Therefore, SCI and the abnormal discharge of brain neurons producing seizure might be closely related [14]. However, KA follows towards the lower parts and will cause rapid stiffness of the lower limbs and tail, producing paraplegia (paralysis of the lower extremities). The KA (1 mL, 0.1 mM) was diluted with 1 mL normal saline to produce a final concentration of 0.05 mM KA, of which 40 µL was injected at the rate of 0.01 mL (10 µL)/min until the syringe was empty. Dura mater is the outermost meningeal membrane covering spinal cord that is composed of collagen fibres and laminas. The needle was inserted 1 mm deep to penetrate into spinal cord parenchymal at the thoracic level as the thickness of dura mater is approximately 0.8 mm for rats [15] (Fig. 4). The lower limbs and tail stiffness that followed rapidly after the injection demonstrated successful injury induction (supplementory video 3). The animal was placed in a prone position for 3–5 min after the KA injection to prevent drug perfusion to the brain, and then gently placed in a supine position on the heating pad. Then, gentle pressure was applied with a surgical sponge to halt the bleeding, taking care not to apply pressure to the spinal cord.

### Wound closure

The muscle layer was carefully sutured over the spinal cord, taking care not to disrupt or apply pressure on the spinal cord. The skin was closed over the wound using 2/0 sutures and the animal was kept on the RodVent ventilator and heating pad until it regained consciousness (60–90 min). Starting from the subcutaneous incision until wound closure the whole procedure took around 20 min. Subsequently, 3–4 mL Ringer lactate solution/300–400 g body weight (pre-warmed to 37 °C) was injected subcutaneously to avoid dehydration due to the surgery. Tramadol stock solution 50 mg/mL was prepared and 0.4 mg/100 g of Tramadol was administered subcutaneously 5 min after wound closure. Povidone and topical antibacterial ointment was applied to the wound. The animal was closely monitored until it regained consciousness, then it was transferred to a regular clean cage with comfortable and clean bedding.

### Postoperative care

After the spinal injury, 0.4 mg/100 g subcutaneous tramadol twice daily for 3–5 days was administered to alleviate pain symptoms. Soft food and autoclaved clean water was provided nearby and easily accessible to the animal. The animal’s daily food and water intake was monitored carefully, and if the animal did not feed and drink properly, Ringer lactate solution (1 mL/100 g body weight subcutaneously) was administered 3–5 days post-injury. Urinary retention typically occurs because of lower limb paralysis. To avoid this, the bladder was manually massaged twice daily to facilitate urination: the abdomen was gently palpated to locate the bladder, and then gentle downward pressure was applied until the bladder was empty. In case of bloody urination, the antibiotic Baytril (100 mg/mL) at 50 mg/100 g body weight was injected subcutaneously.

### Locomotor evaluation and haematoxylin–eosin (H&E) staining

**Fig. 3:**
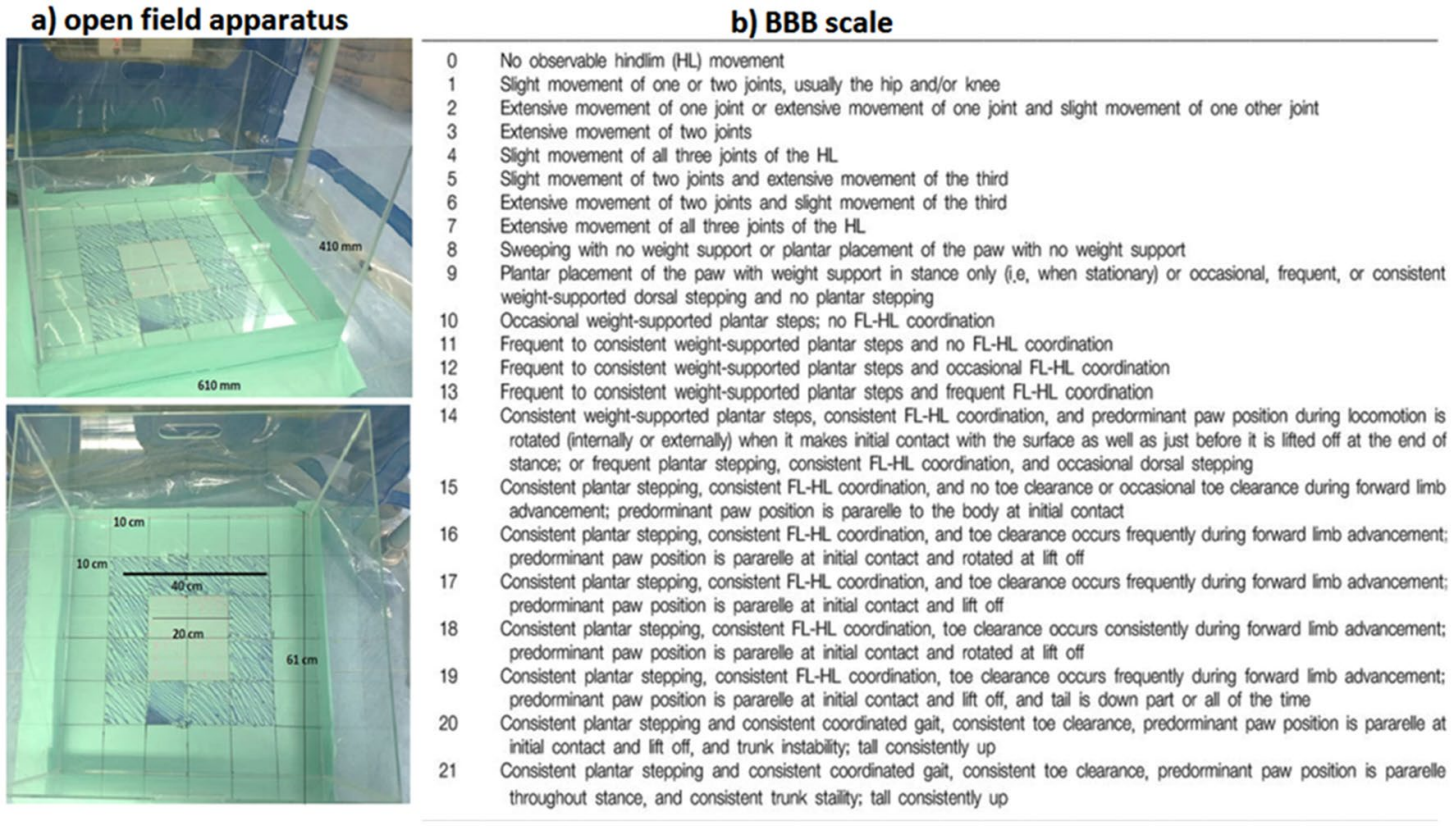
**a** Open field apparatus and **b** Basso, Beattie and Bresnahan (BBB) scale (0–21) to access locomotor and gait analysis of control and KA injured group

Locomotor gait analysis was estimated in the open field apparatus; this apparatus consist of transparent acrylic glass box (610 mm × 410 mm, 5 mm thickness) with floor of the open field is divided into squares (10 cm × 10 cm). The gait and locomotor activity was measured using Basso, Beattie, and Bresnahan (BBB) scale (Fig. 3). The movement of the KA-injured animal was compared with that of control (healthy) rats and was scored by two blinded observer using BBB scale at day 7, 14, 21, and 28 post-injury (Fig. 5). The BBB scale (0–21) represents sequential recovery stages and categorizes combinations of rat joint movement, hind-limb movements, stepping, forelimb and hind-limb coordination, trunk position and stability, paw placement and tail position (Fig. 3). After 28 days of gait analysis, the animals were sacrificed by intraperitoneal administration of 2 mL of pentobarbital (200 mg/mL) as drug overdose [16] and the spinal cord was extracted and placed in 4% paraformaldehyde. The tissue was post-fixed in the solution for 24 h at 4 °C, and then processed with an automatic tissue processor for 14 h. The processed spinal tissue was paraffin-blocked then sliced with a microtome to 5-µm thickness. The slices were fixed on glass slides and dried overnight at 40 °C before being stained with H&E in an automated staining machine. H&E staining was performed to visualise the pathological conditions as compared to that of uninjured spinal cord. The stained slides were mounted and dried overnight at room temperature, then observed under an Olympus digital light microscope.

### Statistical analysis

The BBB scores and other locomotor assessments were evaluated by one-way analysis of variance (ANOVA) for parametric data followed by followed by the Mann–Whitney test. Data are reported as means ± standard deviation and probability values of less than 5% were considered to be significant. All statistical analyses were performed using the Statistical software package running on a compatible personal computer.

## Results

**Fig. 4:**
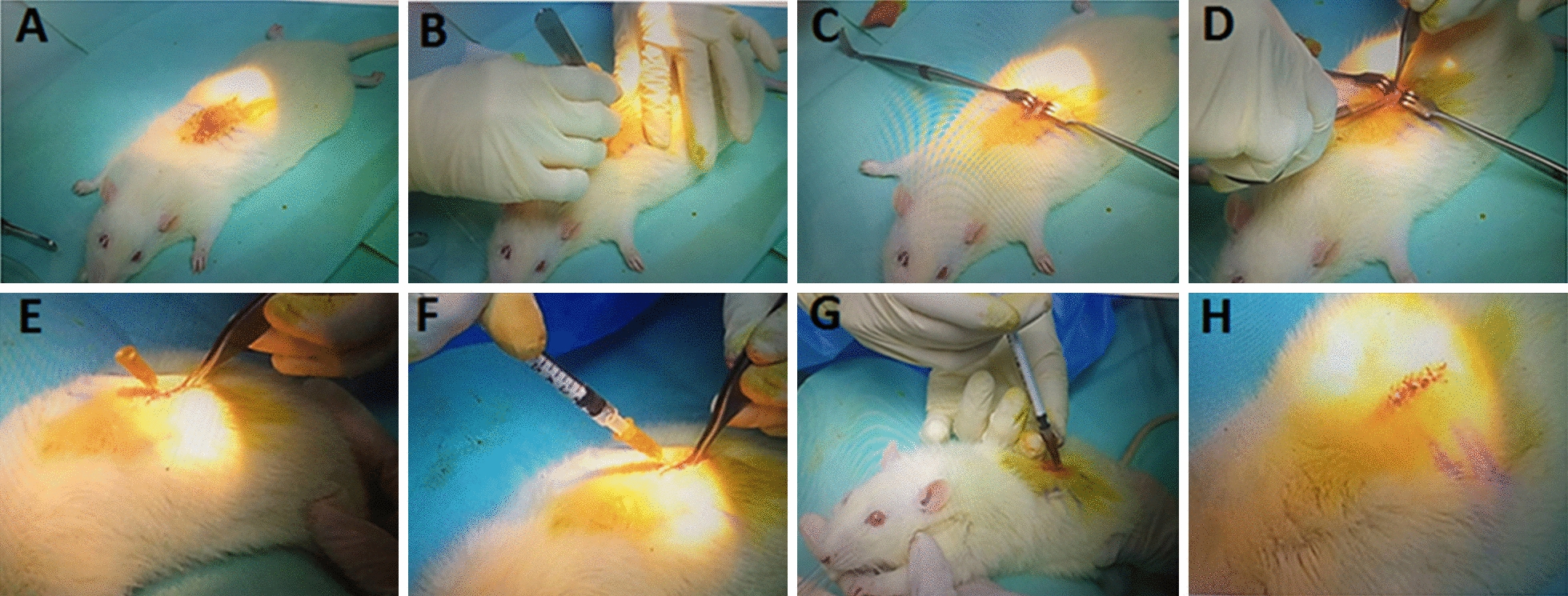
Diagrammatic representation of KA-induced spinal injury. **A** Marking of T11–T13 vertebrae. **B** Subcutaneous incision. **C** Exposure of muscle and spine. **D** Removal of the muscle layer. **E** Insertion of 26-G syringe in vertebral space between T12 and T13 vertebrae. **F** Syringe placement at 45°. **G** Slow perfusion of KA (0.1 µL/min). **H** Wound closure

KA excitotoxicity was induced in five rats [group 1, five healthy rats; group 2, five chemically injured rats]. An important aspect was the KA dose injected: a dose > 50 µL (10 µL/100 g)produced seizures while a dose < 20 µL/100 g failed to induce paraplegia. During the pilot study, 1 mL 0.1 mMKA was injected intra-spinally and the animal developed seizures immediately after the injection and died. Hence, we found that the dose of 10 µL/100 g (0.05 mM)body weight successfully induced paraplegia and selected it for further injury induction. Also during pilot study the sham group (intraspinal injection of normal saline) showed the BBB score similar with the healthy animal, hence only healthy animal were taken as control group. Another important aspect was the rate of KA injection: it was recommended to perfuse KA at the slowest rate to avoid drug penetration to the brain; otherwise, it would result in seizures instead of producing paraplegia. Hence, KA was administered at a rate of 0.01 mL (10 µL)/min. The complete dose of 0.03–0.04 mL (30–40 µL)was administered slowly in approximately 5 min and the needle was placed in the spinal cord tissue for 1 min after KA administration to facilitate drug perfusion. The rate of KA administration was ensured by injecting 10 µL of solution every min in small multiple boluses. Figure 4 depicts the diagrammatic representation.

**Fig. 5:**
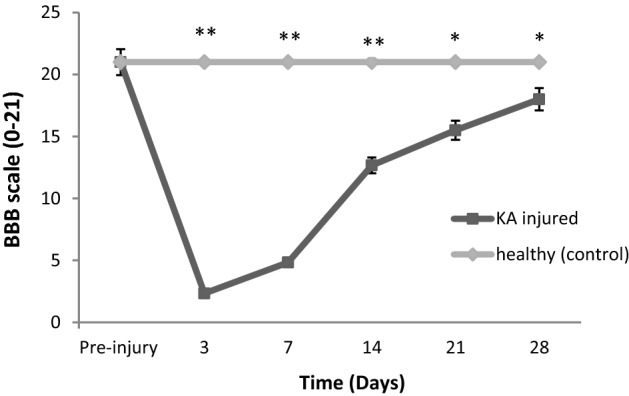
BBB locomotor rating scale scores after KA injury. Data are reported as means ± SD for each group (n = 5), where 0 represent complete paralysis and 21 showed normal gaits. **P ≤ 0.005 for KA injured group and control group at 3, 7 & 14 days post injury and *P ≤ 0.05 for the injured group compared to the control group at 21, & 28 day post injury. Pre injury and control group did not show any significance (ANOVA followed by the Mann–Whitney test). *KA* kainic acid injured rats, *BBB*: Basso, Beattie and Bresnahan, (n = 5)

KA successfully induced injury in the rats; each group contained five rats (KA-treated or uninjured rats) (Fig. 5). The extent of the injury occurred after the injection site throughout the spine tissue. The animal did not move for 3 days and then slowly started to move within 7 days. The motor and sensory alterations in animal behaviour were evaluated using the BBB locomotor rating scale (0–21 points). The KA injured animal showed paralysis of hind-limb and loss of coordination, for first 7 days post injury the KA injured group **P ≤ 0.005 significance were observed in comparison with healthy (control group) at 7 and 14 days, while *P ≤ 0.05 significance were observed between KA injured and control group after 21 and 28 days. The pre-injury and control group did not show any significance (Fig. 5).

In post-operative care of rats, general observation and animal care such as bladder and urine function, water and food intake were formally monitored 3–4 times a day for first 3 days as it is very critical for animal then monitored twice daily up to 7 post-operative days. Each animal was assessed on six categories (body weight, physical appearance, behaviour/activity, clinical signs, lesions/autonomy, and bladder functions. As the bladder management is a very important consideration, the bladder was emptied manually after animal was anaesthetized, prior to SCI [16]. Then immediately after KA injury, as the rats were unable to spontaneously void the bladder was manually expressed within 30 min and the manual expression of bladders (crede) were carried out twice daily until reflex bladder contractions re-emerged after 5–7 days. If the bladder did not empty readily in response to manual expression, then prevention of bladder-related complications was controlled preoperatively with antibiotic prophylaxis. In order to minimize the possibility of rupture, bladder expression must be conducted with patience and care [16].

Besides that, pressure ulceration was also observed on the injured rats, typically on the abdomen, hips, and hind legs. The pressure sores appeared as smooth hairless, red, and inflamed patches. The pressure ulcer or sores were minimized by providing large and clean cages to facilitate movement and kept the animals dry. The cages were cleaned and corn-cob bedding was changed every consecutive day to avoid pressure sores. Rats with SCI were inspected for early signs of pressure sores during daily monitoring; if hairless patches were observed, the amount of bedding was increased, which usually resolved the problem. If redness, inflammation, or skin ulcerations developed, the area was washed (with wound care) and treated with topical antibiotic ointment twice daily until healed [16]. Another important monitoring criterion observed to evaluate animal was the presence or absence of porphyrin accumulation around the eyes. The porphyrin is a reddish discharge produced by the harderian gland in rats and mice. Usually rats overproduce porphyrin when they are stressed and in pain [16].

**Fig. 6:**
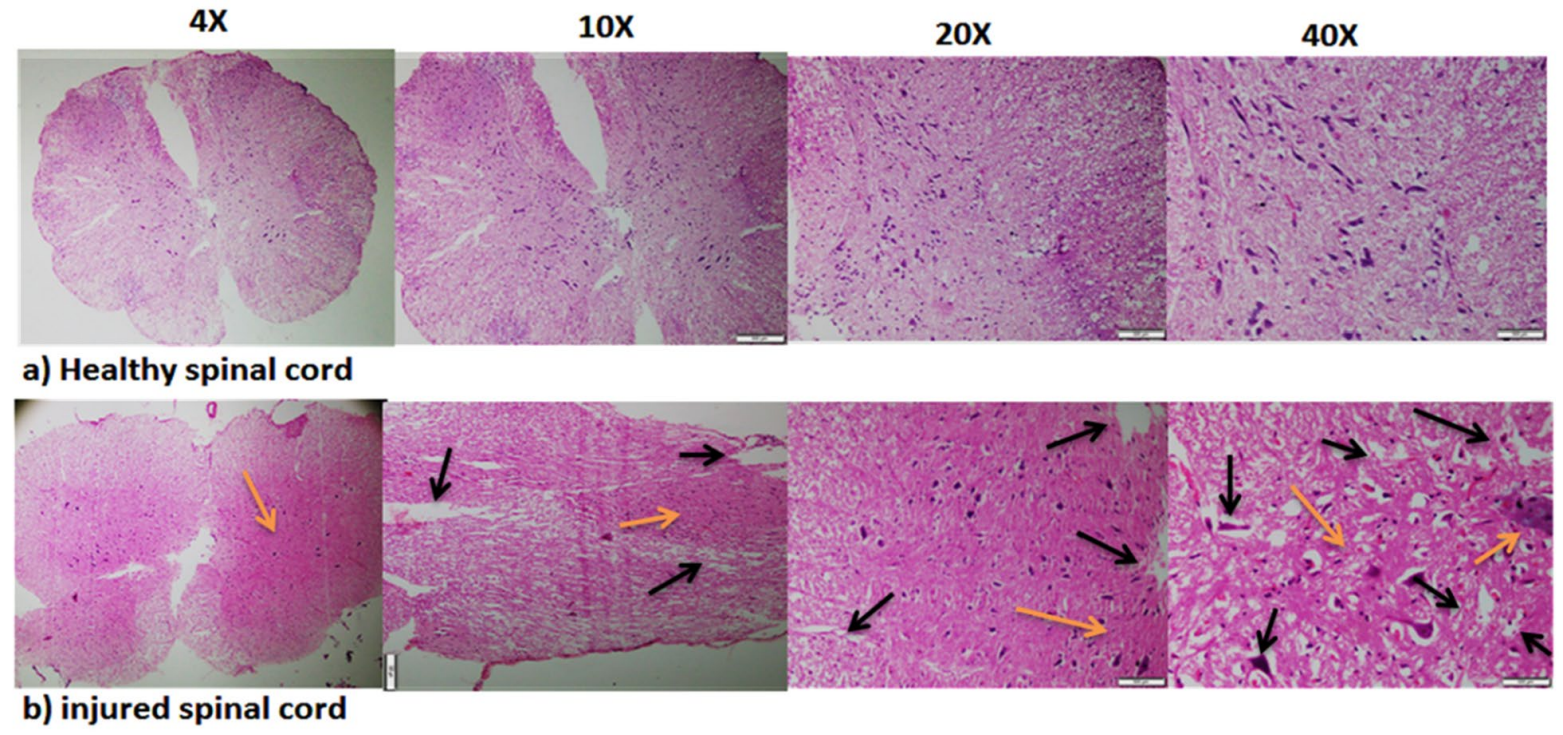
H&E staining demonstrating the morphology of **A** healthy spinal cord sections and **B** chemically injured spinal cord sections at 4×, 10×, 20×, and 40× magnification, Scale bar = 500 µm, n = 5, black arrow indicating cavity formation and orange arrow showed albumin extravasation and revascularization in KA injured tissue

The animals were sacrificed at 28 days post-injury. The spinal cords of the KA-injured animals were fragile and broke easily during extraction as compared to the healthy spinal tissue. The tissue slices were observed under 10×, 20×, and 40× magnification (Fig. 6). The microscopic analysis demonstrated highly distorted grey matter organisation and persistent cavitation in the KA-injured animals. H&E staining of the transverse spinal cord sections showed that group 1 had prominent multipolar neurons with clear nucleus and nucleolus, whereas group 2 exhibited few neurons with loss of nucleolus and nucleic pyknosis in the grey matter, with large neutrophil vacuolation around the damaged neurons. The sections through the centre of the lesion contained numerous haemorrhage spots and visible tissue damage mainly in the central grey matter. The area of tissue occupied by the lesion (visibly damaged tissue and haemorrhage) was outlined and measured (ImageJ, NIH). Moreover, there was more prominent neuronal damage in the ventral grey horn in group 2 (Fig. 6). A significant albumin extravasation was also observed in the KA-injured spinal cord and the increase in albumin extravasation caused revascularization, which is caused by earlier destruction of small blood vessels. The albumin extravasation is directly associated with neuronal damage. The force exerted due to albumin extravasation destroys not only neural cells (neurons, astrocytes, and oligodendrocytes) but also blood vessels around the injury, causing revascularization [17]. During extraction of the vertebrae process, the spinal cord obtained from the KA injured group was observed to be very difficult to remove as an intact spinal cord, whereas the healthy spinal cord tissue remained intact.

## Discussion

The selection of the appropriate model to induce SCI is a very critical aspect [5]. A model producing with pathological features corresponding to humans and simple and easy in induction would be more valuable to study [18]. In the ideal situation animal model should demonstrate these characteristics such as relevance to human pathophysiology, reproducibility, availability, and potential to generate various severities of spinal injury [19]. SCI-based research requires a highly efficient and easily reproducible animal model, which can help in limiting inconclusive data [18]. The traditional method of inducing injury requires different impactors such as New York University [18], Infinite horizon [13], Ohio State University [20], Air gun impactor etc. which are extremely expensive and their availability is limited [5]. Contrary other laboratory-based methods e.g. compression methods such as forceps, clips, and balloon compression methods require personal expertise and are critical in term of reproducibility, which can result in higher mortality rate [5]. In contrast, chemically induced injuries methods are less fatal, simple, and can be accomplished by a variety of chemicals and techniques [21]. The KA-excitotoxicity method used in this research is reproducible, as the amount of drug per animal body weight remains the same, producing similar outcomes. Furthermore, this method enables the determination of postoperative care and the evaluation of treatment options using diverse behavioural and locomotor tests, such as the BBB scale, and sensory function such as the hot spatula test and ethanol cold sensation test [7]. The chemical injury method created similar paraplegia as compared to conventional injury models at very low cost, making it as the most convenient and favourable laboratory method [7]. The KA injury method provides room to evaluate and compare the efficacy of treatments administered during SCI investigative studies, achieving the basic benchmark for an animal SCI model in rating therapeutics [1]. With proper training and practice, the KA-induced SCI method is well-suited in SD rats, which will mimic similar clinical features to that in other animals [22]. Similarly, KA-induced excitotoxicity in rats can be an excellent model for studying the mechanisms of neurodegenerative pathways induced by excitatory neurotransmitters [22]. This technique is highly reproducible and by optimizing the concentration and rate of KA injection, the impairment severity degree can be adjusted, therefore changing the functional and morphological outcomes [6]. However this procedure require skilful personal as the amount and rate of KA administration is very critical, small negligence can stake animal’s life. Thus, a pilot study utilizing 2–3 rats is recommended.

KA is a non-degradable analogue of glutamate and exhibits 30-fold more potent neurotoxicity than glutamate. Intravenous, transcutaneous intrathecal, intraperitoneal, or intranasal injections or microinjection of KA into the amygdala or hippocampus and spinal tissue (intraspinal) cause seizure activity [11] in addition to paraplegia. However, the seizure activity can be prevented by optimising the KA injection dose and rate of administration. Intraventricular injection of 0.5 nM KA in SD rats caused hippocampus rostral pole degeneration, whereas a higher dose, i.e., 4 mM KA, induced neuronal loss [11]. Intra spinal drug administeration was selected because in the intrathecal drug delivery, the drug is introduced to the spinal fluid (intrathecal space), which can increase the chances of recurrent seizures [23]. It was also found that transcutaneous intrathecal administration of excitotoxic agent caused cardiovascular disorders in rats [23]. Whereas, intra-spinal drug delivery delivered excitotoxic drug directly to their site of action, hence the more drug are bound to kainate receptor when drugs are administered intraspinally. Injury at lumber region tend to cause symptoms that are commonly restricted to the tail, but this injury also causes severe bladder dysfunction [24]. However, in SD rats, intra-spinal injection of KA was followed by signs of motor dysfunction and sensory impairment. Behavioural changes in the form of paraplegia (supplementory video 1), limited movement, anxiety, and a tendency to remain seated at the corner of the cage were observed in rats receiving KA [25, 26]. However, seizure activity was not prominently observed in the present study as low KA doses [0.05 mM KA, of which 40 µL was injected at the slow rate of 0.01 mL (10 µL)/min] [25]. To ensure the correct location of injection, needle was inserted approximately 1 mm deep in spinal cord and the reflex was observed in lower part of body, that indicated that the syringe needle penetrated into spinal cord parenchymal rather than in CSF & ISF fluid. Previous study also have shown that if the needle remain in the fluid, there was no reflex and once the needle penetrated into spinal cord parenchymal then only reflex in lower limbs and tail was observed [14]. However seizures or epilepsy may occur due to efflux pathway activation (outflow of drug into perivascular & paravascular space) that acts as crucial facilitator of fluid inflow and outflow thorough neural tissue into ISF & CSF. Only a few animal had uncontrolled muscle contractions (supplementory video 2). Skeletal muscle contraction depends on neuromuscular transmission via spinal motor neuron activation. In vivo and in vitro studies confirmed that motor neurons were highly susceptible to chronic AMPA/kainate receptor-mediated injury [26, 27]. Thus, neuromuscular transmission could not proceed when the spinal motor neurons were injured by KA. Kainate-mediated neuronal excitation was also described as an important factor in the mechanism of other neurodegenerative disorders such as ALS and temporal lobe epilepsy [14].

The H&E results also suggested that SCI by KA administration damaged albumin extravasation and caused inflammation and apoptosis, which were associated with SCI and its pathological changes [14]. The H&E staining slides showed that more cavity formation at lateral and ventral horn than at dorsal horn. The dorsal horn is comprised of sensory nuclei that receive and process incoming somatosensory information while the ventral horn usually comprises of more motor neurons that innervate skeletal muscle [28]. However, this method also has several limitation and challenges such as respiratory arrest associated with seizure that may increase the mortality rate. To overcome this situation, animals were closely monitored and kept under oxygen supported ventilator until animals gain full consciousness. Only mild to moderate spinal cord injury (incomplete) was induced by this KA concentration reflecting the biggest limitation of this method, which is the complete loss of coordination between brain and lower limbs was not observed. Some dragging movement and uncoordinated stepping was observed after 1 week post injury, indicating only mild to moderate SCI was induced by this protocol (supplementory video 1). In order to create severe or complete SCI further study need to be conducted utilizing other potential concentrations of KA (Additional files 1, 2, 3) .

## Conclusion

The excitotoxic model of SCI reflects the morphological changes relative to those described following ischemic and contusion injury in rats and the cascading pathological events are similar to that in humans. Hence, the chemical excitotoxic model demonstrates a clinically pertinent strategy for understanding not only the pathophysiology of SCI but also the underlying apoptotic, degenerative, and regenerative mechanisms. The intra-spinal KA administration induced excitotoxicity, successfully produced paraplegia and alteration of the behavioural responses of the motor neurons below the injured area. Our study demonstrated that KA-induced excitotoxicity is a major component of acute SCI responses that can be correlated with behavioural changes. The proportion of injury induction was observed more than 80% of the five KA-injured rats. Among the 5 animals that were injured by KA, 4 animals completely lost their lower limb movement for 3 days, while 1 animal showed retarded movement. Thus, the success rate of this protocol method was calculated as 80%. The KA excitotoxicity SCI model reflects the onset of spontaneous and evoked abnormal sensations, behavioural changes, and decrease in mobility providing the opportunity for future studies related to abnormal locomotor activity following spinal injury. However other higher concentration of KA could be considered to induce complete tetraplegia and severe paraplegia.

## Supplementary Information

**Additional file 1.** Supporting video 1: Video 1: The gait of KA injured rats 3 days after injury, indicating successful induction of paraplegia (Hindlimbs paralysis).

**Additional file 2.** Supporting video 2: The ocurence of seizure following KA injured rats, within 5 min post injury, showing Myoclonic seizures: a type of seizure that cause a quick, uncontrollable muscle movement with no change in your level of awareness or consciousness.

**Additional file 3.** Supporting video 3: The tail rigidity and mild seizure after KA induced SCI model.

## Data Availability

The data that support the findings of this study are available from the corresponding author upon request.
